# BMC Genetics reviewer acknowledgement 2015

**DOI:** 10.1186/s12863-016-0347-8

**Published:** 2016-02-10

**Authors:** Julia Simundza

**Affiliations:** BioMed Central, 233 Spring Street, New York, NY 10013-1578 USA

## Abstract

The editors of *BMC Genetics* would like to thank all our reviewers who have contributed to the journal in Volume 16 (2015).

**Adam Lukaszewski**

USA

**Vishal Acharya**

India

**Vera Afreixo**

Portugal

**Jon Ahlinder**

Sweden

**Kwangmi Ahn**

USA

**Omar Albagha**

UK

**Florian Alberto**

France

**John Alcorn**

USA

**Emil Alexov**

USA

**Kristina Allen-Brady**

USA

**Santos Alonso**

Spain

**Stefan Ambs**

USA

**Satyanarayana Ande**

USA

**Magnus Andersson**

Finland

**Niki Antypa**

Netherlands

**Juan Jose Arranz**

Spain

**Jennifer Asimit**

UK

**John Attia**

Australia

**Antonios Augustinos**

Greece

**Frédéric Austerlitz**

France

**Jeremy Austin**

Australia

**David Aylor**

USA

**Hela Azaiez**

USA

**Yvonne Badke**

Belgium

**Ernest Bailey**

USA

**Diane Bailleul**

France

**Lorenzo Barchi**

Italy

**Esther Becker**

UK

**Albano Beja-Pereira**

Portugal

**Daniele Bellavia**

Italy

**Olga Beltcheva**

Bulgaria

**Rosaria Benedetti**

Italy

**Tillmann Benfey**

Canada

**Brian Bennett**

USA

**Kerin Bentley**

USA

**Peer Berg**

Norway

**Justo Lorenzo Bermejo**

Germany

**Donagh Berry**

Ireland

**Nils-Ove Bertholdsson**

Sweden

**Viviana Berthoud**

USA

**Etienne Bezault**

USA

**Maria Assunta Biscotti**

Italy

**Didier Boichard**

USA

**Folmer Bokma**

Sweden

**Carolina Bonilla**

UK

**Marina Bonin**

Brazil

**Lara Bossini-Castillo**

UK

**Aniek C. Bouwman**

Netherlands

**Carine Brouat**

France

**Louise Brousseau**

France

**Jácome Bruges-Armas**

Portugal

**Su-Hong Bu**

China

**Bart Buitenhuis**

Denmark

**Cynthia Bulik**

USA

**Jarek Burczyk**

Poland

**Lei Cai**

China

**Yafei Cai**

China

**Mario Calus**

Netherlands

**Xiaoyi Cao**

USA

**Eduardo Casas**

USA

**Sergi Castellano**

Germany

**Hugo Ten Cate**

Netherlands

**Carlos Celis-Morales**

UK

**Lisa Chakrabarti**

UK

**Ken Chalmers**

USA

**Hua Chen**

China

**Hong Chen**

China

**Charles Y. Chen**

USA

**Jianli Chen**

USA

**Piedu Chen**

China

**Wei-Min Chen**

USA

**Yizhou Chen**

Australia

**Vikram Chhatre**

USA

**Yoon Shin Cho**

South Korea

**Bruno Conti**

USA

**Ernest Gus Cothran**

USA

**Eliecer Coto**

Spain

**Vincent Coustham**

France

**Nigel Crawford**

USA

**Marion Cremer**

Germany

**Cheryl Cropp**

USA

**Yuehua Cui**

USA

**Song Cui**

USA

**Tatiana Danilova**

USA

**Andrew Davie**

UK

**M. Davis**

USA

**Tamara Davis**

USA

**Michelle Daya**

South Africa

**Mariza De Andrade**

USA

**Maria Luisa Dettori**

Italy

**H.S. Dhaliwal**

India

**Xianmin Diao**

China

**Alessandro Didonna**

USA

**Mark Dopson**

Sweden

**Mehmet Tevfik Dorak**

UK

**Sami Dridi**

USA

**Britt Drögemöller**

Canada

**Qingzhang Du**

China

**Naomi Duijvesteijn**

Netherlands

**Yann Dussert**

France

**Craig Echt**

USA

**Vahid Edriss**

Denmark

**John Edwards**

USA

**Darrell Ellsworth**

USA

**Rachel Ellsworth**

USA

**Ingegerd Elvers**

USA

**Malena Erbe**

Germany

**Bruno Fady**

France

**Robert Feil**

France

**Bingjian Feng**

USA

**Jane Ferguson**

USA

**Jose Fernandez**

USA

**Susana Ferreira**

Portugal

**Martin Ferris**

USA

**Martin Flajshans**

Czech Republic

**Burkhardt Flemer**

Ireland

**Richard Fletcher**

USA

**Carlos Flores**

Spain

**Jia Nee Foo**

Singapore

**Marina Fortes**

Australia

**Giuliana Fortunato**

Italy

**Breno Fragomeni**

USA

**Wenqing Fu**

USA

**Maria Fuciarelli**

Italy

**Silvia Fuselli**

Italy

**Agata Gadaleta**

Italy

**Andrea Gaedigk**

USA

**Mercedes Garayoa**

Spain

**Christiane Gebhardt**

Germany

**Phil Giffard**

Australia

**Elizabeth Gilbert**

USA

**Tania Gioia**

Germany

**Ian Gizer**

USA

**Andreas Gogol-Doering**

Germany

**Pengtao Gong**

China

**George Goulielmos**

Greece

**Manje Gowda**

India

**Surendra Goyal**

India

**Paul Grabowski**

USA

**Cecile Grenier**

Colombia

**Ben Greyling**

South Africa

**Thomas Gridley**

USA

**Darren Griffin**

UK

**Daniel Grinberg**

USA

**Vince Kornél Grolmusz**

Hungary

**Christine Große-Brinkhaus**

Germany

**David Groth**

Australia

**Xing-You Gu**

USA

**Rudy Guerra**

USA

**Carlos Guerrero-Bosagna**

Sweden

**Paul Gugger**

USA

**Longbiao Guo**

China

**Yunqian Guo**

China

**Shicheng Guo**

USA

**Sanjeev Gupta**

Ireland

**Jenny Hagenblad**

Sweden

**M Haile-Mariam**

Australia

**David Haldane**

Canada

**Jianlin Han**

Kenya

**Lee Harold**

USA

**Liang He**

Finland

**Meian He**

China

**Xinhua He**

China

**Xin He**

USA

**Tao He**

USA

**Scott Hebbring**

USA

**Chew Kiat Heng**

Singapore

**Julie Hicks**

USA

**André Hidalgo**

Netherlands

**Emmeline Hill**

Ireland

**Katrin Hinderhofer**

Germany

**Lies Hoefsloot**

Netherlands

**Gabriel Hoffman**

USA

**Josephine Hoh**

USA

**Daniela Holtgräwe**

Germany

**Ian Hope**

UK

**Jian Hou**

China

**Liping Hou**

USA

**Guo Hu**

China

**Jie Hu**

USA

**Yijuan Hu**

USA

**Xu Hua**

China

**Lei Huang**

China

**Wei Huang**

China

**Kai Huang**

USA

**Hailiang Huang**

USA

**Wen Huang**

USA

**David Hughes**

Spain

**Stefanie Huhn**

Germany

**György Hutvágner**

Australia

**Kamal Ibrahim**

USA

**Meryem Ipek**

Turkey

**Daniel Irimia**

USA

**Brian Irish**

USA

**Akira Ishikawa**

Japan

**Jeanne Jacobs**

New Zealand

**Malgorzata Jankun**

Poland

**Luc Janss**

Denmark

**Yulin Jia**

USA

**Xueqiu Jian**

USA

**Bruce Johnson**

USA

**Jenny Juengel**

New Zealand

**Haja Kadarmideen**

Denmark

**Lukáš Kalous**

Czech Republic

**Guolian Kang**

USA

**Juha Kantanen**

Finland

**Rebecca Kartzinel**

USA

**Jaebum Kim**

Republic Of Korea

**Jung Han Kim**

USA

**Elizabeth King**

USA

**Rick Kittles**

USA

**Christoph Knorr**

Germany

**Michael Hans Kohn**

USA

**Caline Koh-Tan**

UK

**Roland Kölliker**

Switzerland

**Willem Kruijer**

Netherlands

**Haydn Kuchel**

Australia

**Santosh Kumar**

Canada

**Jaya Kumari**

Norway

**Vanisha Lakhina**

USA

**Denis Laloe**

France

**Hovirag Lancioni**

Italy

**Nicholas Larson**

USA

**Sabrina Le Cam**

France

**Ju-Seog Lee**

USA

**Seunggeun Lee**

USA

**Sigrid Lehnert**

Australia

**Gabriel Leitner**

Israel

**Johannes Lenstra**

Netherlands

**Chun Li**

China

**Ao Li**

China

**Hui Li**

China

**Zhang Li**

China

**Qigang Li**

China

**Zitong Li**

Finland

**Jun Li**

USA

**Zichao Li**

China

**Yuqing Li**

USA

**Yi Li**

Singapore

**Xiaoxi Lin**

Canada

**Xu Lin**

China

**Gabriella Lindgren**

Sweden

**Amanda Lindholm-Perry**

USA

**Mugen Liu**

China

**Xu Liu**

China

**Bin Liu**

China

**Jianquan Liu**

China

**Wansheng Liu**

USA

**Zengting Liu**

Germany

**Adrian Llerena**

Spain

**Stephanie London**

USA

**Saioa Lopez**

Spain

**Siew Low**

Australia

**Lizhi Lu**

China

**Xinhong Luan**

China

**Pierre Luisi**

Spain

**Adam Lukaszewski**

USA

**Keming Luo**

China

**Ma Luo**

Canada

**Jiangtao Luo**

USA

**Guansheng Ma**

China

**Xiaochun Ma**

China

**Peipei Ma**

China

**Steffen Maak**

Germany

**Margaret Mackinnon**

Kenya

**Panagiotis Madesis**

Greece

**Laurence Maréchal-Drouard**

France

**Vladmir Margarido**

Brazil

**Osvaldo Marinotti**

USA

**Donata Marletta**

Italy

**Kathleen Martin**

USA

**Cesar Martins**

Brazil

**Scot Matkovich**

USA

**Taylor Maxwell**

USA

**James Mccreight**

USA

**Molly Mccue**

USA

**Michael Mcgeachie**

USA

**J. Mitchell Mcgrath**

USA

**Kieran Meade**

Ireland

**Jennifer Meadows**

Sweden

**Hendrik-Jan Megens**

Netherlands

**Andreas Menke**

Germany

**Susan Meyer**

USA

**Phil Miklas**

USA

**Dan Milbourne**

Ireland

**Cecelia Miles**

USA

**Gabriel Minarik**

USA

**Juan Miranda-Rios**

Mexico

**Suresh Mishra**

Canada

**Xavier Montagutelli**

France

**Christopher Moran**

Australia

**Gota Morota**

USA

**Jeong-Hwan Mun**

South Korea

**Steven Munger**

USA

**Moses Muraya**

Germany

**Hideki Mutai**

Japan

**Teruyoshi Nagamitsu**

Japan

**Hugo Naya**

Uruguay

**Christopher Nelson**

UK

**Frank Nicholas**

Australia

**Garth Nicholson**

Australia

**Motohide Nishio**

Japan

**Dan Nonneman**

USA

**Johannes Novak**

Austria

**Domenico Nuzzo**

Italy

**Thomas O'Brien**

USA

**Jorgen Odegard**

Norway

**Thomas Odong**

Netherlands

**Junko Okano**

Japan

**Patricia Okubara**

USA

**Jose L. Oliver**

Spain

**Angélica Olivo-Díaz**

Mexico

**Ettore Olmo**

Italy

**Hanne Gro Olsen**

Norway

**Bode Olukolu**

USA

**Akio Onogi**

Japan

**Tamás Orbán**

Hungary

**Pablo Orozco-Terwengel**

UK

**Richard Orrell**

UK

**Wei Pan**

USA

**Manish Pandey**

India

**Bibhusita Pani**

USA

**Afshin Parsa**

USA

**Hemal Patel**

USA

**Hubert Pausch**

Germany

**Michele Pazzola**

Italy

**Jason Peart**

Australia

**Jill Pecon-Slattery**

USA

**Niels Pedersen**

USA

**Francisco Peñagaricano**

USA

**Hans-Peter Piepho**

Germany

**Marija Pljesa-Ercegovac**

Serbia

**Andrea Porceddu**

Italy

**Manoj Prasad**

India

**Marcin Pszczola**

Poland

**Deirdre C Purfield**

Ireland

**Xinshuai Qi**

USA

**Wanqiong Qiao**

USA

**Padmalatha Rai**

India

**G Ravikanth**

India

**Sarah Rea**

Australia

**James Reecy**

USA

**Monika Reißmann**

Germany

**Romdhane Rekaya**

USA

**Jun Ren**

China

**Pascal Rihet**

France

**David Riley**

USA

**Isabel Rivera**

Portugal

**James Roberds**

USA

**Christèle Robert-Granié**

France

**Dale Roberts**

Australia

**Monica Rodriguez**

Italy

**Juan Rodriguez-Flores**

USA

**Claire Rogel-Gaillard**

France

**Megan Rolf**

USA

**Zsolt Ronai**

Hungary

**Stephanie Rosse**

USA

**Florence Rouleux-Bonnin**

France

**Yuefeng Ruan**

Canada

**Sachin Rustgi**

USA

**Peter Ryan**

Australia

**Mahdi Saatchi**

USA

**Francois Sabot**

France

**Giuseppe Saccone**

Italy

**Wolfgang Sadee**

USA

**Goutam Sahana**

Denmark

**Srinivas Vinod Saladi**

USA

**Lucas Sanchez**

Spain

**Juliano Sangalli**

Brazil

**Miguel Santana**

USA

**Pratik Satya**

India

**Ana Savic-Radojevic**

Serbia

**Ruairidh Sawers**

Mexico

**Ian Sayers**

UK

**Joseph Schacherer**

France

**Flávio Schenkel**

Canada

**Antje Schierholt**

Germany

**Christian Schlötterer**

Austria

**Jennifer Schmidt**

USA

**Armin Schmitt**

Italy

**Andreas Schmitz**

Switzerland

**Robert Schmitz**

USA

**Dustin Schones**

USA

**Britta Schulz**

Germany

**Hervé Seligmann**

France

**Nick Serão**

USA

**Andrey Shcherban**

Russian Federation

**Amir Sherman**

Israel

**Clive Shiff**

USA

**Masayoshi Shigyo**

Japan

**Kenta Shirasawa**

Japan

**Mukerjee Shomita**

India

**Daniel Shriner**

USA

**Chang Shu**

USA

**Animesh Shukla**

USA

**Sanjeev Shukla**

India

**Timothy Shutt**

Canada

**Katja Silbermayr**

Austria

**Xueling Sim**

Singapore

**Magda Sindicic**

Croatia

**Shree Ram Singh**

USA

**Johanna Sistonen**

Switzerland

**Loren Skow**

USA

**Tony Slater**

Australia

**Petr Smykal**

Czech Republic

**Eric Snyder**

USA

**Leah Solberg Woods**

USA

**Qingxuan Song**

USA

**Chi Song**

USA

**Pavel Souček**

Czech Republic

**Leon Spicer**

USA

**James Squires**

Canada

**Brendan Stamper**

USA

**Charles Steward**

UK

**Paul Stothard**

Canada

**Patrick Stratz**

Germany

**Guosheng Su**

Denmark

**Kazuhiko Sugimoto**

Japan

**G Suman Latha**

India

**Fanyue Sun**

USA

**Tatiana Suslova**

Russian Federation

**Nicolas Sylvius**

UK

**Joanna Szyda**

Poland

**Hidenori Tachida**

Japan

**Kobi Tadmor**

Israel

**Fasil Tekola-Ayele**

USA

**Raimund Tenhaken**

Austria

**Jens Tetens**

Germany

**Sophie Thevenon**

France

**Vicki Thomson**

Australia

**Francesco Tiezzi**

USA

**Peter Tiffin**

USA

**Alex Todorov**

USA

**Motoshi Tomita**

Japan

**Eleni Tsiplakou**

Greece

**Matthew Tucker**

Australia

**Christopher Tuggle**

USA

**Jennifer Tullet**

UK

**Jasim Uddin**

Australia

**Yoshinobu Uemoto**

Japan

**Giampiero Valè**

Italy

**Armand Valsesia**

USA

**Pim Van Hooft**

Netherlands

**Paul Vanraden**

USA

**Roel Veerkamp**

Netherlands

**Fabio Veronesi**

Italy

**Frédérique Viard**

France

**Marcelo Vicari**

Brazil

**Carlos Vicient**

Spain

**Joseph Vinetz**

USA

**Christof Vulsteke**

Belgium

**Patrik Waldmann**

Sweden

**Stefan Walter**

USA

**Kathryn Walters-Conte**

USA

**Ming-Bo Wang**

Australia

**Chaolong Wang**

Singapore

**Yaqun Wang**

USA

**Tao Wang**

USA

**Ning Wang**

China

**Tao Wang**

USA

**David Warrilow**

Australia

**Jonas Warringer**

Sweden

**Markus Weber**

Switzerland

**Karen Weck**

USA

**Zhi Wei**

USA

**Changshuai Wei**

USA

**Jochen Weishaupt**

Germany

**Emanuel Weitschek**

Italy

**Jared Westbrook**

USA

**Stephen White**

USA

**Kenneth Witwer**

USA

**Weiren Wu**

China

**Xifeng Wu**

USA

**Zheng Xia**

USA

**Yan Xiang**

China

**Zengyan Xie**

China

**Chao Xing**

USA

**Lizhen Xu**

Canada

**Yan Xu**

China

**Yong Xu**

China

**Haiming Xu**

China

**Jun Xu**

USA

**Shawn Xu**

USA

**Lingyang Xu**

USA

**Peng Xu**

China

**Cheng Xue**

USA

**José Yáñez**

Chile

**Bing Yang**

USA

**Daichang Yang**

China

**Shengping Yang**

USA

**Chao-Yie Yang**

USA

**Yao Yang**

USA

**Guoyou Ye**

Australia

**Bin Yi**

China

**Jiye Yin**

China

**Hunter Young**

USA

**Neil Youngson**

Australia

**Bing Yu**

USA

**Paolo Zambonelli**

Italy

**Mehdi Zarrei**

Canada

**Weiwei Zhai**

Singapore

**Wenguang Zhang**

China

**Liangsheng Zhang**

China

**Fengyu Zhang**

China

**Deqiang Zhang**

China

**Yuan-Ming Zhang**

China

**Peng Zhang**

USA

**Wei Zhang**

USA

**Xinyu Zhang**

USA

**Xiquan Zhang**

China

**Tongwu Zhang**

USA

**Xueyong Zhang**

China

**Yong Zhang**

China

**M Zhang**

China

**Chen Zhao**

China

**Ni Zhao**

USA

**Jinshun Zhao**

China

**Min Zhong**

USA

**Xiang Zhou**

USA

**Ling Zhou**

China

**Jun Zhu**

China

**Jieyun Zhuang**

China

